# Cracking the Code: Investigating the Correlation between Aerobic Vaginitis and Preterm Labor

**DOI:** 10.3390/medicina60040648

**Published:** 2024-04-18

**Authors:** Panagiota Zarmakoupi, Alexandros Psarris, Christina Karasmani, Panagiotis Antsaklis, Marianna Theodora, Michael Syndos, Andreas Pampanos, Kalliopi I. Pappa, Ekaterini Domali, Nikolaos Thomakos, Karolina Akinosoglou, Aristotelis Tsiakalos, George Daskalakis

**Affiliations:** 1 1st Department of Obstetrics and Gynecology, Alexandra Hospital, 11527 Athens, Greece; p.zarmakoupi@gail.com (P.Z.); psarris.alexandros@gmail.com (A.P.); panosant@gmail.com (P.A.); martheodr@gmail.com (M.T.); sindosgyn@hotmail.com (M.S.); apampanos@yahoo.com (A.P.); kpappa@med.uoa.gr (K.I.P.); kdomali@yahoo.fr (E.D.); thomakir@hotmail.com (N.T.); gdaskalakis@yahoo.com (G.D.); 2 Department of Internal Medicine and Infectious Diseases, Medical School University of Patras, 26504 Patras, Greece; akin@upatras.gr; 3 Leto General, Maternity & Gynecology Clinic, 11524 Athens, Greece; atsiakalos@gmail.com

**Keywords:** aerobic vaginitis, desquamative aerobic vaginitis, pregnancy outcomes, preterm labor

## Abstract

Aerobic vaginitis (AV) is a distinct clinical entity characterized by inflammation and abnormal vaginal microflora. Often mistaken for bacterial vaginosis, AV remains relatively unknown and underdiagnosed. AV’s understanding is evolving, with some experts suggesting it may primarily be an immunological disorder, the prevalence of which has a range of 7–13% in non-pregnant women and 4.1–8.3% during pregnancy. Pregnancy can affect susceptibility to vaginal infections, leading to adverse outcomes for the woman and the newborn. This review summarizes the correlation between AV and adverse pregnancy outcomes, particularly preterm birth, the leading cause of morbidity and mortality among neonates. An improved understanding of AV’s impact on pregnancy outcomes can lead to early recognition, proper management, and effective interventions. While some studies support an association between AV and preterm labor, the existing knowledge of this relationship remains limited. The evidence suggests that AV may contribute to adverse pregnancy outcomes, mainly preterm birth, but further research is needed to establish a definitive link. Further studies are needed to investigate the underlying mechanisms and clarify AV’s role in premature labor. A comprehensive understanding of AV’s impact on pregnancy outcomes is crucial for early recognition, appropriate management, and effective interventions.

## 1. Introduction

Aerobic vaginitis (AV) is a distinct vaginal infectious entity characterized by an overgrowth of commensal aerobic microorganisms, mostly of intestinal origin. This condition gives rise to an abnormal (dysbiotic) vaginal microflora consisting of aerobic, enteric bacteria, variable levels of vaginal inflammation, reduced numbers of lactobacilli, and deficient epithelial maturation [1].

The exact nature of the relationship between AV and the disrupted vaginal microflora, which is predominantly aerobic, is still not fully understood. While some consider AV a strict bacterial infection, others propose it is an immunological disorder that influences vaginal microflora, or a dermatological disease in the vagina [2]. This intriguing perspective highlights the multifaceted nature of AV, warranting further investigation into its underlying mechanisms. In the state of health, the vaginal microbiota comprises a dynamic ecosystem consisting of diverse microbial populations, present in varying proportions, operating to safeguard the vaginal epithelium against different infectious agents.

Symbiotic bacteria, particularly *Lactobacillus* species, play a crucial role in maintaining vaginal health. These bacteria are responsible for the acidic pH of the vagina, produced through the conversion of glycogen stored in epithelial cells into lactic acid. Lactobacilli form a protective layer on the vaginal mucosa, preventing the adhesion and penetration of pathogens. Furthermore, they produce hydrogen peroxide, bacteriocins, and bacteriocin-like substances, reinforcing the mechanisms against invasion and colonization by opportunistic pathogens [3,4].

Pregnancy induces alterations in the immune response, potentially influencing susceptibility to vaginal infections. Studies have found that estrogen reduces the resistance of vaginal epithelial cells to pathogens, while progesterone enhances the adhesion of pathogens to the vaginal epithelium [5,6]. Moreover, estrogen and progesterone may increase the virulence of pathogenic strains, potentially leading to severe adverse outcomes. Approximately 25–40% of preterm deliveries are linked to infections, either directly or indirectly through inflammation processes. The pathways leading to preterm labor involve the activation of specific biochemical mechanisms, including increased genital tract prostaglandins and proteases, progesterone withdrawal, and changes in hormone concentrations. Inflammatory responses may also contribute to the preterm premature rupture of membranes (PPROMs). Inflammation, typically a regulatory mechanism for tissue response, can result in an exaggerated immune response, with an increased production of inflammatory cytokines, elastases, and matrix metalloproteinases (MMPs). This heightened inflammation triggers the withdrawal of progesterone, crucial for maintaining pregnancy.

This review aims to explore the correlation between AV and adverse pregnancy outcomes, focusing on preterm birth. Preterm delivery (PTD), defined as delivery before 37 weeks of gestation by the WHO, is a significant global public health issue [7,8]. Neonatal morbidity and mortality present significant challenges, particularly in developed countries, where they serve as primary determinants of adverse outcomes and globally stand as the second leading cause of death among children under five years old [9]. This study seeks to contribute to the knowledge we have of reproductive health by rigorously examining the potential correlation between AV and adverse pregnancy outcomes. By unraveling the complexities of such interactions, the present study seeks to establish a strong basis for promptly identifying potential risks associated with AV during pregnancy. The knowledge gained from this investigation is intended to guide timely and evidence-based interventions, thereby fostering the development of precise and effective management strategies. Through these efforts, the ultimate goal is to enhance maternal well-being during pregnancy and, consequently, reduce the incidences of neonatal morbidity and mortality.

## 2. Materials and Methods

**Figure 1:**
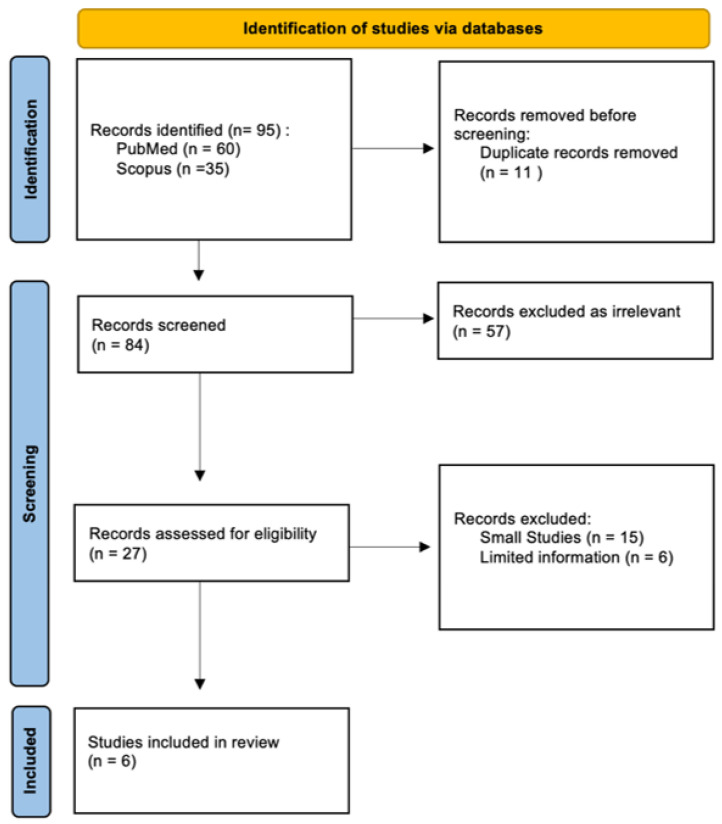
Process of elimination and inclusion of studies for review. After conducting a PubMed and Scopus search, a total of 95 articles were reviewed, out of which 11 were removed as duplicates, 57 were deemed irrelevant, and 21 were excluded due to limited information and small sample sizes. Finally, 6 studies were deemed suitable and included in the final review. PRISMA flow diagram [10].

A comprehensive literature review using PubMed and Scopus databases (January 2002 until October 2022) was conducted. The specific query employed was (“aerobic vaginitis” OR “DIV” OR “desquamative inflammatory vaginitis”) AND (“pregnancy outcomes” OR “preterm delivery” OR “preterm birth” OR “preterm labor” OR “premature delivery”), cross- referencing the resulting articles with the query: aerobic vaginitis preterm birth. In our review, we included original research articles and review articles published in English from 2002 to 2022, based on searched keywords from the databases of PubMed and Scopus, considering the correlation between aerobic vaginitis and preterm labor. Our study excluded non-English studies, conference abstracts, letters to the editor, and ex vivo studies. A total of 95 articles were identified from the databases. Initially, 11 articles were removed as duplicates. The abstracts were screened for relevance, which allowed us to exclude 57 articles that did not discuss any form of AV, but other forms of vaginitis, or did not address pregnancy. After careful analysis and exclusion of 21 more studies, which did not include a sufficient number of participants or did not approach the investigated relationship between AV and preterm birth, 6 studies were included in the final review (Figure 1). Three independent researchers reviewed the articles and hand-searched literature. Disagreements were discussed and resolved.

## 3. Results

In this article, we investigated the correlation between AV and PTB across seven different studies (Table 1). Among these studies, five provided evidence supporting the correlation between AV and PTB, while two could not prove the claim.

The first study [11], conducted by Donders et al. in 2009, involved 759 pregnant women at 9–16 weeks out of 1026 women who were requested to participate in the sampling procedure. The results indicate that AV associated to the presence of cocci microflora in the microscopic examination, also known as coccoid AV, is associated with increased risks of extremely preterm births (OR 3.2; 95% CI 1.2–9.1) and miscarriages (OR 5.2; 95% CI 1.5–17). Similar are the results of the second study by Donders et al. in 2010 [12]. This study referred to the same population of 1026 low-risk pregnant women before the 16th week as the first study conducted by Donders et al. After the analysis, it was determined that women with severe AV at 10–14 weeks had shorter cervix lengths at 20–24 and 30–34 weeks, which increased the likelihood of preterm birth. AV severity is defined with both clinical and microscopic criteria. The typical clinical picture includes an erythematosus vaginal mucosa, a pH higher than 6, sticky discharge, and odor. As for the microscopic criteria, the standard scoring of vaginal specimens examined by a phase contrast microscope varied from 0 to 10, as Donders et al. introduced [1] for AV-severity categorization (Table 2). The combination of the five following parameters (lactobacillary grades, number of leucocytes, proportion of toxic leukocytes and parabasal cells, and type of background flora) led to a composite score. A score of less than 3 stands for no AV, 3–4 for light AV, 5–6 for moderate AV, and severe AV is defined as a score ≥6. Severe AV is identical to the entity that is called desquamative inflammatory vaginitis (DIV) and stands for a severe chronic clinical syndrome. Na Li’s study [13], which involved 685 pregnant women at 22.3 ± 8.6 weeks, revealed that the incidences of preterm birth, premature rupture of membranes, neonatal jaundice, and neonatal infection were significantly higher in the AV group compared to the control group (*p* < 0.001, <0.001, =0.007, and =0.025). Additionally, Mahmoud F. Hassan et al.’s study [14] on 600 34–36-week pregnant women showed a significant correlation between preterm birth and AV (*p* = 0.001). Cha Han et al.’s study [15], which included 624 pregnant women and 365 non-pregnant women in the third trimester, found a correlation between AV and the premature rupture of membranes (PROMs) (*p* = 0.003), even though the difference was not significant regarding PTB (*p* = 0.236).

On the other hand, two studies did not yield positive results regarding the correlation between AV and PTB. Krauss Silva et al.’s study [16], comprising 1199 pregnant women, <20 weeks gestation, without risk factors, with a pH > 4.5, did not show an association between moderate AV and spontaneous preterm delivery or abortion. As limitations of the study, we should consider the fact that the analyzed samples were subsamples of Gram-stained vaginal smears with an intermediate-degree infection and BV, and less than 5% presented moderate AV with no severe cases. The use of Gram staining resulted in the exclusion of the proportion of toxic leukocyte overstimulation of the occurrence of disturbances of vaginal flora, making the diagnosis questionable.

Furthermore, in Thi Cha Nguyen et al.’s study [17] on 323 pregnant women in the third trimester, comprising 84% with light and 16% with moderate AV, did not observe any effect of AV on either PROMs or preterm premature rupture of membranes (pPROMs). The study revealed that a significant majority of women (84%) displayed a moderate type of asymptomatic BV without any instances of severe AV. Moreover, the occurrences of *E. coli* and *S. agalactiae* bacteria were notably lower compared to previous research. The incidence of preterm births was also minimal in both groups studied. However, the study’s weakness lies in its sample size calculation, tailored mainly to identify AV incidence in pregnant women. Consequently, the sample size might be insufficient to highlight the differences in outcomes beyond AV incidence effectively.

## 4. Discussion

Our review provides contradictory evidence found in the global literature regarding the association of AV and PTB. Even though most studies presented here support the correlation between abnormal vaginal flora and preterm birth, it is important to note that existing literature also includes studies that lack evidence of a positive association. These findings emphasize the complexity of this relationship and the need for further research to understand AV’s impact on adverse pregnancy outcomes.

AV was initially identified by the Belgian scholar Donders and colleagues in 2002, and its prevalence varies across regions. While the prevalence of AV in non-pregnant women is in the range of 4.2–25.8%, it is estimated to be 4.1–8.3% during pregnancy. The most frequently encountered pathogens, including *E. coli*, *Klebsiella pneumoniae*, *Staphylococcus aureus*, *group B Streptococcus (GBS)*, and *Enterococcus faecalis*, appear in varying proportions among several studies [18,19]. The diagnosis of AV relies on wet mount microscopy, performed by a phase-contrast microscope, in order to assess lactobacillus grades and other variables, such as leukocytes, the percentage of toxic leukocytes and parabasal epithelial cells, and the type of background flora. All the parameters mentioned above serve as parts of a composite score that leads to the final diagnosis [1,20].

As stated above, AV is relatively unknown and underdiagnosed, and commonly mistaken for BV (bacterial vaginosis). The features that contribute to the differential diagnosis of BV are listed in the table below (Table 3) [3,4].

While some studies support an association between aerobic vaginitis (AV) and preterm labor, the existing knowledge of this relationship remains limited. The available evidence, from the limited number of concrete studies included in our review, suggests that AV may contribute to adverse pregnancy outcomes, particularly preterm birth, but further research is needed to establish a definitive link.

A significant percentage (25–40%) of preterm deliveries can be attributed to various pathogens causing infections directly (overt or subclinical) and indirectly via the inflammation process elicited by such infections [21]. The pathological processes that lead to preterm labor involve the activation of one or more components of a specific biochemical pathway, such as the increased production of prostaglandins and proteases in the genital tract, the functional withdrawal of progesterone, and changes in hormone concentrations, such as the corticotropin-releasing factor (CRF) and cortisol [22,23,24]. These inflammatory processes may also lead to the preterm premature rupture of membranes (PPROMs) [25].

Inflammation can be considered as a regulative mechanism by which the tissues respond to injurious stimuli in order to control and repair possible damage. More specifically, inflammation induces an exaggerated immune response that increases the production of inflammatory cytokines, elastases, and matrix metalloproteinases (MMPs), and triggers the functional withdrawal of progesterone, a vital hormone for pregnancy’s maintenance [26,27].

Regarding infections, bacteria are found in the fetal circulation in 30% of intra-amniotic infections, leading to a systemic inflammatory response in the fetus [25]. The most common route of infection is ascending [28], while there is also a possibility of hematogenous dispersion with transplacental passage [29]. Most likely, pathways for infection-induced PTB include decidual stimulation and the fetal immunological response, both as innate immune system reactions [21,30]. Moreover, a fetal response takes place as the infection promotes the release of the corticotropin-releasing hormone and, subsequently, the release of fetal corticotropin and fetal cortisol from both the placenta and the fetal hypothalamus, resulting in prostaglandin production [31].

As supporting evidence, it is worth considering two more studies that suggest a correlation between abnormal vaginal flora and PB. Szubert [3] examined 396 pregnant women with an abundant growth of aerobic bacteria and fungi, where 413 had physiologic vaginal biocenosis, and found a significantly higher percentage of PB among patients infected with GBS than those not infected, while there was no statistical correlation between PTB and *Klebsiella* spp., *S. aureus*, or *Candida* spp. infection. Meanwhile, Hocevar [32] suggested that preterm relative to term pregnancies have greater richness and diversity of the vaginal microbiome, along with reduced Lactobacillus species.

Supporting our case is also the existing body of knowledge regarding the influence of vaginal infections on pregnancy outcomes, and specifically the impact of the extensively studied BV. In a comprehensive systematic review conducted by Josiane Kenfack-Zanguim in 2022, after an exhaustive search extending from January 2002 until December 2022, 26 articles met the inclusion criteria. This review served as a vital compendium of contemporary knowledge pertaining to the impact of bacterial vaginosis (BV) on maternal and neonatal outcomes.

Among the findings, it was revealed that PTB exhibited the highest prevalence at 17.9%, with a 95% confidence interval ranging from 13% to 23.3% across the various studies. Moreover, the study unveiled a significant association between BV and PTB, with an odds ratio (OR) of 1.76 and a 95% confidence interval from 1.32 to 2.35. Additionally, BV was also linked to the premature rupture of membranes (PROMs) with an OR of 2.59 and a 95% confidence interval from 1.39 to 4.82 [33].

Similarly, Mohanty [34] conducted a meta-analysis, including 20 articles, which produced strikingly consistent results. This meta-analysis also proves a significant association between bacterial vaginosis and preterm birth, with an overall odds ratio (OR) of 1.79 and a 95% confidence interval from 1.32 to 2.43.

Considering the shared pathophysiological mechanisms between BV and AV and the inflammatory factors associated with AV, this entity may be a potential candidate to cause pregnancy complications. Specifically, dysbiosis occurs in both AV and BV, leading to a decrease in lactobacillus-dominated microflora, disrupting the healthy vaginal ecosystem. However, AV presents a unique profile, marked by a more pronounced increase in interleukin 1β (IL-1β) compared to BV, combined with elevated levels of interleukin 6 (IL-6) and interleukin 8 (IL-8), which are not commonly seen in BV cases [35,36,37]. Both AV and BV involve the production of sialidases, enzymes that diminish the local immune response [36,38]. And this disturbance in the regulation of local immunity appears to be crucial in the development of AV. More specifically, an imbalance in local immune regulation has been observed in women with aerobic vaginitis (AV). In AV, there is a significant increase in IL-1β levels, reaching 178.8 pg/mL, compared to bacterial vaginosis (BV) with 71.2 pg/mL and normal controls with 5.0 pg/mL (*p* < 0.001) [37]. Additionally, there is a notable elevation in IL-6 and IL-8, although this increase in pro-inflammatory cytokines is not consistently observed in BV, as noted by most researchers [38], though some dissenting opinions exist [36]. This surge in cytokines appears to be linked to a reduction in lactobacilli in the vaginal microbiota [39]. However, it is worth mentioning that a similar decrease in lactobacilli in BV does not provoke a pro-inflammatory response.

Contrary to the theory proposing that microbial sialidases in BV might dampen the cytokine cascade and reduce IL-8 levels, AV shows high IL-8 levels and sialidase activity [36]. IL-6 plays a crucial role as an initiator of the inflammatory process [40], while IL-8 acts as a chemoattractant for neutrophils, promoting their migration and activation in infected areas [37]. Neutrophil response to IL-8 involves the release of granule enzymes, induction of phagocytosis, and various intracellular and extracellular alterations. It is worth noting that the levels of vaginal cytokines, particularly the IL-1 receptor antagonist, may vary between pregnant and non-pregnant women [38]. Certain cytokine levels, including IL-6 and IL-8, may also experience fluctuations during a normal pregnancy. These natural changes during pregnancy should be considered when examining mucosal immune responses to infections of the lower genital tract.

Additionally, AV frequently presents with lower local estrogen levels, a prevalent characteristic, whereas in BV, this is not consistently implicated as an etiological factor [40]. The presence of aerobic bacteria contributes to the pathophysiological pathways in different ways, depending on the particular characteristics of each bacterium [1,41].

*E. coli* is the most frequently cultured Gram (−) microorganism in AV, principally as the only isolated microorganism. Its role is controversial, but it is still one of the most frequent causes of neonatal sepsis and chorioamnionitis [42]. In the study conducted by Lobos et al., *E. coli* in 46 cases was described as the only microorganism isolated in cultures, taken from 425 women with vaginal infections. The genetic subtypes of *E. coli* were the same subgroup in 98% of the cases. The authors suggested that these strains probably constitute a subpopulation within the other species specifically found in vaginal secretions. Although the finding’s clinical implications remain unclear, one can assume that specific subtypes deriving from the vagina may be associated with adverse pregnancy outcomes. The fact that the specific *E. coli* strain is an uropathogen also supports the above-described hypothesis [43].

Undoubtedly, conducting a thorough exploration of treatment options aimed at preventing preterm birth stands as a pivotal endeavor in mitigating the associated complications. The most effective way to treat AV in both pregnant and non-pregnant women remains uncertain. Over the years, several therapeutic approaches have been suggested. Antibiotics, like metronidazole, have been commonly used, but their effectiveness is unclear. Studies show that metronidazole may not decrease the risk of preterm birth in women with AV, and, in some cases, it might even increase the risk. Furthermore, research conducted by Odendaal [44] and Klebanoff [45], focusing on the treatment of trichomoniasis or bacterial vaginosis (BV), revealed a higher likelihood of preterm births following metronidazole treatment. This finding has led several experts to reach a strong consensus that using metronidazole during pregnancy to mitigate the risk of preterm births should be avoided.

On the other hand, when researchers employed antibiotics with a broader spectrum, capable of targeting Gram-positive cocci and E. coli, they achieved a decrease in the incidence of preterm births in the majority of placebo-controlled studies, such as oral clindamycin [46]. Although not all of them showed the same positive results, for example the use of vaginal clindamycin in the treatment of bacterial vaginosis (BV) by Kekki et al. [47], which indicated that that vaginal clindamycin did not lead to a reduction in the rate of preterm deliveries or peripartum infections when compared to the placebo group. In a study by Tempera et al. [48], local kanamycin was evaluated in non-pregnant women with AV. Through a thorough analysis of culture results, the researchers suggested that this topical treatment could be an effective method for addressing Enterobacteriaceae in the context of AV. Although some of these options for the use of antibiotics seem to be very promising, testing during pregnancy with such agents has not been conducted in a systematic way in order to provide us with robust data.

The use of probiotics has also been reported, mainly to treat abnormal vaginal flora during pregnancy, and not specifically as a regime for AV. A Cochrane review of all randomized controlled trials assessing the prevention of preterm birth through the use of antibiotics showed that, while probiotics seem to be effective in treating vaginal infections during pregnancy, there is currently a lack of adequate trial data to evaluate their impact on preterm birth and its associated complications [49]. We should also consider the fact that AV can co-occur with other genital tract infections, such as BV, candidiasis, and sexually transmitted infections; therefore, the therapy must be tailored to each patient. In the studies analyzed, no therapeutic agent was used, so the development of research to clarify the appropriate treatment regimen seems of utmost importance.

Our review bears a number of limitations, including the heterogeneity of the studies reviewed in terms of the population under study, the gestational age, the diagnosis criteria, and further particular characteristics of each study. To address this limitation, future research endeavors should be meticulously designed, ensuring an adequate number of participants and adopting a standardized methodology. This approach will facilitate meta-analyses, allowing for a comprehensive synthesis of findings across studies and enabling more robust conclusions to be drawn.

Additional studies are necessary to define the contours of AV’s impact on adverse pregnancy outcomes, focusing on preterm labor. Gaining a thorough understanding of how AV influences the outcome of pregnancy holds significant importance for early recognition, suitable care, and successful interventions. A comprehensive exploration of the impact of aerobic vaginitis (AV) on adverse pregnancy outcomes, particularly its association with preterm labor, necessitates further studies. The complex effects of AV on pregnancy need further exploration as the current knowledge is insufficient. Understanding the nuanced interactions between AV and pregnancy outcomes holds paramount importance for various reasons. Firstly, it facilitates the early recognition of potential risks, enabling healthcare professionals to intervene promptly. Secondly, it paves the way for the development of suitable care strategies tailored to address the specific challenges posed by AV during pregnancy. Additionally, a thorough comprehension of how AV affects pregnancy outcomes is crucial for the implementation of successful interventions that can positively impact maternal and neonatal health. By advancing our insights into this domain, we are ready to achieve improved reproductive health results, with a particular focus on mitigating the impact of preterm birth. Ultimately, these endeavors contribute to enhancing the overall well-being of both women and neonates, underscoring the importance of continued research in this critical area of maternal and child health. Progressing our insights into this domain will inevitably result in improved reproductive health results, lessening the impact of preterm birth and enhancing the well-being of both women and neonates.

## 5. Limitations of Study

While our study contributes valuable insights into the correlation between aerobic vaginitis and preterm labor, several limitations should be acknowledged. Firstly, our search strategy may have been susceptible to publication bias, as we focused on English-language studies and may have missed relevant research published in other languages or unpublished literature. Additionally, the heterogeneity among the included studies, such as variations in study designs, populations, and definitions of aerobic vaginitis and preterm labor, presents challenges in synthesizing and interpreting the findings. Furthermore, while we aimed to address potential confounding factors, such as age, parity, and socioeconomic status, in our analysis, unresolved issues may still exist. Finally, the generalizability of our findings may be limited to the populations and settings represented in the included studies. Despite these limitations, our study provides a comprehensive overview of the existing literature on this topic and highlights areas for future research.

## Figures and Tables

**Table 1:** Studies investigating the correlation between AV (aerobic vaginitis) and PTB (preterm birth).

Study	Patient Inclusion Criteria	AV Diagnostic Criteria	Severity	Gestational Age at Screening	Outcome	OR/*p*-Value
Donders et al. (2009) [11]	759 pregnant women	Donders’ modified score		9–16 weeks	Coccoid AV associated with increased risks of EPTB and miscarriage	EPTB OR 3.2; 95% CI 1.2–9.1/ OR 5.2; 95% CI 1.5–17)
Donders et al. (2010) [12]	1026 low-risk pregnant women	Donders’ modified score	Severe	Before 16 w	Severe AV at 10–14 weeks; the cervix appeared shorter at 20–24 and at 30–34 weeks than in other women	
Na Li et al. [13]	685 pregnant women (control group *n* = 503, AV group *n* = 182)	Donders’ modified score		22.3 ± 8.6 weeks	Incidences of pb, PROM, neonatal jaundice, and neonatal infection significantly higher in the AV group	<0.001 =0.007, =0.025
Mahmoud F. Hassan et al. (2020) [14]	600 pregnant women	Composite AV score ≥ 3 determined by saline wet mount microscopy		34–36 weeks	PTB	0.001
Cha Han et al. [15]	624 pregnant women and 365 on-pregnant women	Score > 3 for saline wet mount microscopy		3rd trimester	PROM: 0.003, but no PTB: 0.236	0.003
Krauss-Silva et al. [16]	1199 pregnant women without risk factors, pH > 4.5	Donders’ modified score (proportion of toxic leukocytes criterion was not considered in the modified score used)		<20 weeks of gestation	No spontaneous case of PD or abortion was associated with severe or moderate AV	
Thi Chau Nguyen et al. [17]	323 pregnant women	Donders’ modified score and culture	84% light, 16% moderate	3rd trimester	Puerperal sepsis 6%, Pb rate low for both groups (2% vs. 3.3%); no effect of AV on either PROM or pPROM	0.02

**Table 2:** Donders’ modified score: the diagnostic criteria involve the combination of AV (aerobic vaginitis) Gram staining and clinical manifestations [1]. (LBGs: lactobacillary grades, PBCs: parabasal epitheliocytes).

Score	LBG	Number of Leukocytes	Proportion of Toxic Leukocytes	Background Flora	Proportion of PBC
0	I and IIa	<10/hpf	None or sporadic	Unremarkable or cytolysis	None or <1%
1	IIb	>10/hpf and ≤10/epithelial cell	≤50% of leucocytes	Small coliform bacilli	≤10%
2	III	>10/epithelial cell	>50% of leucocytes	Cocci or chains	>10%

**Table 3:** Differential diagnosis between aerobic vaginitis (AV) and bacterial vaginosis (BV).

	AV	BV
Bacteria	*E.coli*, *Klebsiella pneumoniae*, *E. faecalis*, *S. aureus*, Coagulase negative, GBS	*Gardnerella vaginalis*, *Prevotella* spp., *Mycoplasma*, *Mobiluncus* spp., *Peptostreptococcus*
Inflammation	Present, often severe	Absent
Discharge	Yellowish, sticky	White to gray, thick, homogenous
Odor	Foul, rotten	Fishy, especially when reacting with KOH
Symptoms	Increased leucorrhoea, possible dyspareunia	Increased leucorrhea, itching
PH	>5.5	>4.5
KOH test	Negative	Positive

## Data Availability

The data used to support the findings of this study are included in the article.
